# Highly pathogenic avian influenza A(H5N1) virus infection on multiple fur farms in the South and Central Ostrobothnia regions of Finland, July 2023

**DOI:** 10.2807/1560-7917.ES.2023.28.31.2300400

**Published:** 2023-08-03

**Authors:** Erika Lindh, Hanna Lounela, Niina Ikonen, Tuija Kantala, Carita Savolainen-Kopra, Ari Kauppinen, Pamela Österlund, Lauri Kareinen, Anna Katz, Tiina Nokireki, Jari Jalava, Laura London, Marjaana Pitkäpaasi, Jaana Vuolle, Anna-Liisa Punto-Luoma, Riikka Kaarto, Liina Voutilainen, Riikka Holopainen, Laura Kalin-Mänttäri, Terhi Laaksonen, Hannu Kiviranta, Aino Pennanen, Otto Helve, Ilona Laamanen, Merit Melin, Niina Tammiranta, Ruska Rimhanen-Finne, Tuija Gadd, Mika Salminen

**Affiliations:** 1 Finnish Institute for Health and Welfare – THL, Helsinki, Finland; 2 Finnish Food Authority, Helsinki, Finland; 3 Finnish Food Authority, Seinäjoki, Finland

**Keywords:** Avian influenza, outbreak, mink, fox, raccoon dog, fur animal farms, HPAI, H5N1, Finland, mammalian infection, highly pathogenic avian influenza

## Abstract

Since mid-July 2023, an outbreak caused by highly pathogenic avian influenza A(H5N1) virus clade 2.3.4.4b genotype BB is ongoing among farmed animals in South and Central Ostrobothnia, Finland. Infections in foxes, American minks and raccoon dogs have been confirmed on 20 farms. Genetic analysis suggests introductions from wild birds scavenging for food in farm areas. Investigations point to direct transmission between animals. While no human infections have been detected, control measures are being implemented to limit spread and human exposure.

Highly pathogenic avian influenza (HPAI) A(H5N1) virus belonging to clade 2.3.4.4b has since the end of April 2023 caused widespread outbreaks in wild and domestic birds in 25 countries in Europe [1]. In wild birds, black-headed gulls have been heavily affected, with mass deaths observed in many places, including in Finland. First reported on 14 July, an outbreak of avian influenza among farmed foxes, minks and raccoon dogs occurred in the regions of South and Central Ostrobothnia and is still ongoing. Up to 27 July, animals on 20 farms have been affected. Here, we provide an initial description of the outbreak and control measures taken, and discuss the source, potential reasons for and consequences of the outbreak.

## Outbreak setting

Several types of animals are commercially farmed for fur production in Finland, including American mink (*Neovison vison*), arctic (blue) fox (*Vulpes lagopus*), red (silver) fox (*V. vulpes*) and their crossbreeds, raccoon dog (*Nyctereutes procyonoides*) and sable (*Martes zibellina*). There are over 500 farms in the country, and 95% of the fur production (1.3 million animals annually) is concentrated in western Finland. Animals are mostly kept in side-by-side wire-mesh cages in narrow raised sheds (shade houses). These are only covered by a roof and have no closed walls. Birds, including gulls and jackdaws, visit the farms and seek access to the feed of the fur animals in the shade houses. Outbreaks of HPAI subtype H5N1 among birds, especially black-headed gulls, with mass wild bird deaths have occurred in multiple regions of Finland during the summer of 2023, as in almost all European Union/European Economic Area countries [1].

## Outbreak description

On 12 July 2023, the Finnish Food Authority (FFA) informed the Finnish Institute for Health and Welfare (THL) about a suspicion of cases of avian influenza among farmed fur animals on five farms in the South and Central Ostrobothnia region of western Finland. Because of rising mortality on the farms, especially in juvenile fur animals, cadavers were sent to the FFA laboratory for analysis. Dead animals exhibited no obvious cause of disease but had lesions in the lungs and signs of septicaemia. Follow-up investigation by FFA revealed symptoms characteristic of HPAI H5N1 virus infection in mammalian species (lethargy, neurological signs, diarrhoea, rapid death) occurring among the fur animals on affected farms. Subsequently, the causative agent was confirmed by PCR at FFA to be HPAI H5N1 virus clade 2.3.4.4b. This is only the second known outbreak of infection by this avian influenza virus variant at fur animal farms in Europe, after one reported in 2022 in Spain [2].

To date, 27 July 2023, 20 affected farms in four municipalities of Central and South Ostrobothnia have been identified (Figure) with blue and silver foxes and their crossbreeds, raccoon dogs and minks being infected. The affected farms, which vary in size from 600–50,000 fur animals, house a total of 37,900 minks, 142,463 foxes and 5,400 raccoon dogs (a total of 185,763 fur animals, each farm rearing 1–3 species).

**Figure:**
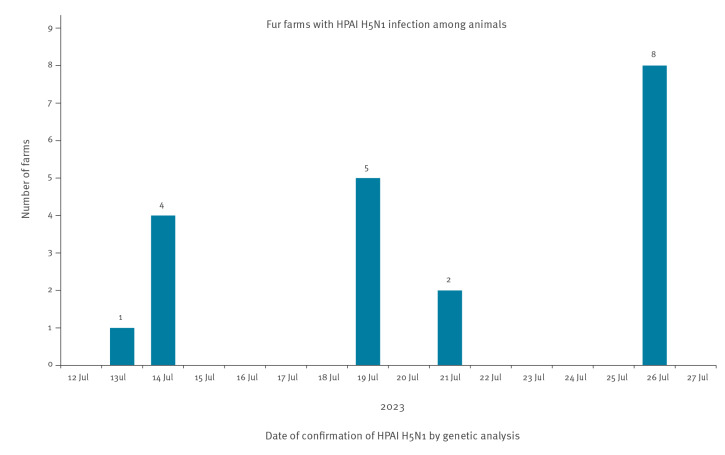
Fur farms with highly pathogenic avian influenza A(H5N1) virus infection among animals^a^, South and Central Ostrobothnia region, Finland, 12–27 July 2023 (n = 20)

^a^ Blue and silver foxes (11 and one farm, respectively) and their crossbreeds, raccoon dogs (one farm) and minks (one farm). Information on species affected pending from the rest of the farms.

Of note, several animal species were infected on two farms (blue foxes and minks on one, silver foxes and raccoon dogs on one).

## Origin of the infection on fur farms

Preliminary results from whole genome sequencing of 14 representative individual fur animal samples from 11 farms (fox n = 11, mink n = 3) and from seven wild black-headed gulls from mostly nearby municipalities in Finland (GISAID accession numbers are provided under Data availability) from the outbreak, confirm that all the HPAI samples from fur animals are BB genotype. BB genotype is a H5N1 virus variant widely found in seagulls all over Europe, and similar to HPAI H5N1 present locally in gulls (data not shown), suggesting that at least the original exposure and transmission stems from exposure to the birds [1]. Phylogenetic analyses further suggest that several introductions from birds to the fur animals may have taken place but are also consistent with possible transmission among the fur animals themselves and potentially even between species. Transmission between fur animals is also supported by the general epidemiological pattern of several hundreds of sick and dead animals on the 20 farms (mortality on affected farms has been 2–4 times the normal rate and, at the peak of the outbreak, a large farm recorded almost 400 deaths in one day, which is 10 times the normal rate). The exact mechanism of the transmission within and between farms is, however, not yet known.

Some evidence for adaptation to replication in mammals is evident, as the PB2 gene E627K change was detected in samples from one farm and the T271A change in a sample from another farm.

## Control measures on fur farms

After the first findings of HPAI H5N1 virus infections on fur farms, the FFA initiated actions and gave instructions for control measures and improvement of biosecurity at the farms (Box).

Box **First measures of control and prevention in response to the highly pathogenic avian influenza A(H5N1) virus outbreak, South and Central Ostrobothnia region, Finland, July 2023**

1. Raising awareness among farmers and veterinarians as well as other stakeholders

2. Promoting reporting of sick animals and mortality changes with a low threshold

3. Strengthening protection against wild birds entering the farm

4. Stopping animal movements off or within the affected farms

5. Culling of sick animals

6. Keeping records of sick and dead animals

7. Safe carcass disposal

8. Recommending no manure removal from the farm

9. Avoiding visits to poultry or pig holdings or other fur farms

10. Improving biosecurity measures for entering or exiting the farm

11. Advising on cleaning and disinfection of facilities

12. Instructions on occupational protection and health measures for the farmers as issued by the Finnish Institute of Occupational Health (TTL)

As more affected farms were detected, the Ministry of Agriculture and Forestry amended the national legislation on 18 July 2023, making it possible for animal health authorities to order restrictions and culling of animals in case HPAI virus infection was detected in fur animals [3]. Previously, this possibility only existed for poultry and captive birds. At some farms, all animals will need to be culled while in others, where the spread of the disease is limited, only animals in affected shade houses will be culled. In the latter cases, the situation will be closely monitored, to ensure the stamping out of the disease.

## Public health measures

The Finnish Institute for Health and Welfare established a situation management team and issued recommendations for PCR testing of humans possibly exposed to avian influenza on the fur farms, mainly farm and culling staff and veterinarians, as well as simultaneous subtyping of any influenza findings [4]. The Finnish Institute for Health and Welfare also has the capacity to rapidly produce sequencing data (either subgenomic or whole genome) from any influenza-positive sample for phylogenetic and mutation analysis, both of human and animal origin. Unrelated to the current fur farm epidemic outside the seasonal influenza period, all influenza A-positive samples from hospitalised patients in Finland are typed, and only seasonal H1N1pdm09 and H3N2 virus strains have been discovered in 2023.

The Finnish Institute for Health and Welfare recommends that contacts of confirmed or probable HPAI-infected fur animals on the farms should seek testing after an incubation period of 6-8 days post-exposure, irrespective of whether they have symptoms compatible with respiratory infection or not. For symptomatic individuals, testing is also recommended to their symptomatic secondary contacts. So far, 32 persons have been tested, three of them with influenza-like symptoms. Up to 1 August 2023, no tested individuals have been positive for either avian or seasonal influenza infection. Testing recommendations are based on European Centre for Disease Prevention and Control (ECDC) guidance [5].

Multiple channels of communication have been used to raise awareness of the currently elevated risk levels among both veterinary and human health professionals, and to inform the public about the HPAI H5N1 virus epidemiology throughout Europe and Finland [6,7].

## Discussion

The HPAI H5N1 outbreak on fur farms in South and Central Ostrobothnia in Finland, which was detected in July 2023, is not over yet. Active control measures appear to be effective, while culling of the animals is underway. The ongoing epizootic of HPAI H5N1 among gulls and other birds poses a risk of re-introduction in the current farm settings. It is clear that current conditions on the majority of farms cannot prevent bird access and much more rigorous biosecurity measures would have to be put in place at the industry level to eliminate these risks.

The sequence data indicate that, at least originally, transmission likely occurred from birds to the fur animals, most probably through contacts in the shade houses. Birds have easy access to the interior of the shade houses and gulls have frequently been observed in the vicinity of the farms. Mass deaths of gulls have also occurred in the same general region. Other potential exposure possibilities have been investigated, such as contamination of fur animal feed by birds or indirect spread by the workers while handling or feeding the fur animals. In addition, direct contacts between farms through personnel or animal movements, have been investigated and excluded as a cause of spread. In theory, an infected human could have transmitted the disease to the animals, but no evidence exists to support such a scenario.

At present, it appears likely that transmission among fur animals is contributing to the evolution of the outbreak, and PB2 mutations associated with improved replication in mammalian cells have been detected in a subset of the fur animal cases. A well-recognised concern exists that prolonged replication of the HPAI H5N1 virus in a high-density mammalian population, such as the fur farms, might lead to viral forms that could more easily spread among humans [8-10]. As there is little prior experience of outbreaks similar to the one described here, it is not possible to predict the outcome. Thus, no firm conclusions can yet be drawn on the current risks for fur animal-to-human or human-to-human transmission.

## Conclusion

No human infections have been detected thus far in the current fur farm outbreak in Finland and, globally, there is no verified transmission of HPAI H5N1 virus infection from another mammal to humans. However, this outbreak does raise concerns for the future, not only in Finland but in the global context. Thus, very rigorous monitoring of the situation at fur farms in Finland is being implemented in close cooperation among national authorities and in consultation with relevant international public health agencies. More detailed analyses of the outbreak are planned to be published as sequencing and potentially also serological data become available.
